# Exploratory evaluation supported by experimental and modeling approaches of *Inula viscosa* root extract as a potent corrosion inhibitor for mild steel in a 1 M HCl solution

**DOI:** 10.1515/biol-2022-0879

**Published:** 2024-07-11

**Authors:** Mohamed Adil Mahraz, Rajae Salim, El Hassania Loukili, Amine Assouguem, Mohammed Kara, Riaz Ullah, Ahmed Bari, Hafize Fidan, Abdelouahid Laftouhi, Amine Mounadi Idrissi, Belkheir Hammouti, Zakia Rais, Mustapha Taleb

**Affiliations:** Laboratory of Engineering, Electrochemistry, Modeling and Environment, Faculty of Sciences Dhar El Mahraz, Sidi Mohamed Ben Abdellah University, Fez 30050, Morocco; Euromed University of Fes, UEMF, Fez, Morocco; Laboratory of Biotechnology, Environment, Agri-Food and Health, Faculty of Sciences Dhar El Mahraz, Sidi Mohamed Ben Abdellah University, Fez 30050, Morocco; Laboratory of Applied and Environmental Chemistry (LCAE), Faculty of Sciences, Mohammed First University, B.P. 717 60000, Oujda, Morocco; Laboratory of Functional Ecology and Environment, Faculty of Sciences and Technology, Sidi Mohamed Ben Abdellah University, PO. Box 2202 Imouzzer Street, Fez 30000, Morocco; Laboratory of Biotechnology, Conservation and Valorisation of Natural Resources (LBCVNR), Faculty of Sciences Dhar El Mahraz, Sidi Mohamed Ben Abdallah University, BP 1796 Atlas, Fez 30000, Morocco; Department of Pharmacognosy, College of Pharmacy, King Saud University, Riyadh, Saudi Arabia; Department of Pharmaceutical Chemistry, College of Pharmacy, King Saud University, Riyadh, Saudi Arabia; Department of Tourism and Culinary Management, Faculty of Economics, University of Food Technologies, Plovdiv, Bulgaria

**Keywords:** *Inula viscosa*, HPLC, corrosion inhibitor, DFT, electrochemical impedance spectroscopy

## Abstract

The corrosion of metals poses a threat to the economy, the environment, and human health due to undesirable reactions and contaminated products. Corrosion inhibitors, including natural products, can play a key role in protecting metallic materials, especially under challenging conditions. In this study, the roots of the *Inula viscosa* plant were examined for their ability to act as corrosion inhibitors in a 1 M hydrochloric acid (HCl) solution. Different extracts of the plant were evaluated for their corrosion inhibition capacity in a 1 M HCl solution. The effectiveness of different plant extracts was assessed, including an aqueous extract, an ethanolic extract, and a combined water–ethanol extract. Compounds present in the roots of *Inula viscosa* were identified using high-performance liquid chromatography. The electrochemical properties of the extracts were studied using various techniques such as open circuit potential, electrochemical impedance spectroscopy, and potentiodynamic polarization. Additionally, surface analysis after immersion was performed using scanning electron microscopy. Electrochemical data revealed that *Inula viscosa* root (IVR) extracts acted as mixed-type corrosion inhibitors with pronounced cathodic characteristics. The inhibitory efficiency was closely related to the concentration of *Inula viscosa* (*I. viscosa*), showing a significant increase with higher concentrations. This resulted in a decrease in corrosion current and an increase in polarization resistance. Notably, inhibitory efficiency reached high levels, up to 97.7% in mixed extract which represents a mixture between water and ethanol. In our study, it was observed that the mixed extract (water + ethanol) allowed for a greater corrosion inhibition compared to the other solvents studied, 97.7%. Surface analyses confirmed the formation of an organic film layer on the steel surface, attributed to the bonding of functional groups and heteroatoms in *I. viscosa* components. Therefore, this study paves the way for the potential integration of *I. viscosa* as a promising corrosion inhibition material, offering durable protection against steel corrosion and opening avenues for various related applications.

## 1 Introduction

Substances like HCl and H_2_SO_4_ are essential in various industrial processes such as cleaning, descaling, pickling, and more [1]. However, in sectors where mild steel (MS) is a major infrastructure, corrosion rates are accelerated in acidic environments, leading to annual losses of billions of dollars. Preventing the contact between acid and metal is of paramount importance. Organic inhibitors, when applied in low concentrations, create a barrier against acid contact, thus reducing corrosion rates [2]. Depending on the chosen extraction or synthesis method, inhibition can take both organic and inorganic forms. It is crucial to consider the inherent properties of these inhibitors. Biodegradability is fundamental, ensuring corrosion inhibitors can naturally degrade through environmental processes, reducing their long-term impact. Non-toxicity is also essential to ensure that inhibitors do not pose risks to human health or result in harmful pollution.

The availability of inhibitors is another critical consideration. Ideally, the materials needed for inhibitor production should be easily accessible, enabling their widespread application across various industries. Furthermore, environmental sustainability is paramount when selecting inhibitors, as minimizing the impact on ecosystems and maintaining a balance between industrial needs and nature preservation is necessary. The importance of considering these factors when selecting and applying corrosion inhibitors cannot be overstated [3,4]. Considering these aspects is essential to ensure effective corrosion protection while minimizing adverse environmental and human health effects. Currently, the market offers a diverse range of corrosion inhibitors, but many come with a high price tag and are highly toxic, posing risks to human health and the environment [5,6]. Therefore, there is an urgent need to develop green corrosion inhibitors that exhibit strong corrosion inhibition effects and are cost-effective, intending to replace the toxic corrosion inhibitors on the market. Addressing this issue has led to increased attention to green corrosion inhibitors. These inhibitors encompass amino acids, proteins, plant extracts, cellulose, starch, and more [7,8,9]. As a result, research into plant-based inhibitors for green corrosion chemistry is gaining popularity. Natural compounds are well-suited to act as acid corrosion inhibitors due to their cost-effectiveness [10,11,12,13]. Organic molecules containing heteroatoms such as N, S, and O tend to be present on the metal surface, adsorbed at active sites, forming a thin protective layer that reduces the passage of corrosive substances through the metal [14,15,16,17]. The components of natural products that adhere to the metal surface are influenced by various factors, including (a) the chemical composition of the inhibitors, (b) the nature of the surface, and (c) the metal’s charge. Compounds derived from plants containing heterocyclic components like steroids, alkaloids, and flavonoids have been studied for their effective corrosion inhibition properties [18,19,20,21]. Numerous studies have been conducted on the use of plant extracts to prevent corrosion [22,23,24,25,26,27,32,33,34,35].

A complex assortment of secondary metabolites has previously been identified in *I. viscosa*, including flavonoids such as sakuranetin, 7-*O*-methylaromadendrine, and 3-*O*-acetylpadmatine, sesquiterpene lactones, acids such as inuviscolide, tomentosine, and their derivatives, carabrone, ilicic acid, costic acid, and their derivatives, as well as phenolic acid derivatives such as caffeoylquinic acid, dicaffeoylquinic acid, and their derivatives, glycolipids such as inugalactolipid A, and triterpenoids [36].

*Inula viscosa* L. (*I. viscosa*) (Asteraceae) is a medicinal plant widely used in traditional medicine. The growth of this plant depends on environmental conditions, including light energy, water, and soil nutrients. The Inula genus comprises more than 100 species [37] and is commonly found in the Mediterranean basin. In Morocco, *Inula viscosa* (L.) Aiton, also known as *Dittrichia viscosa* (L.), locally referred to as “Bageraman,” is a perennial Mediterranean herbaceous plant belonging to the Asteraceae family. It has a history of being used in folk medicine to treat animal wounds and has been employed to address various health concerns in different regions. North African traditional medicine has been used to address diabetes and inflammation [38]. In Jordan, it has been utilized to treat tuberculosis anemia and as a poultice for rheumatic pains [39]. It has been known in Spain for its antiseptic properties, for managing skin inflammations, and for treating gastro-duodenal disorders [40,41]. Scientific research conducted on this plant has demonstrated its high antioxidant, antimicrobial, and antifungal activity [42,43,44,45,46,47].

Research on corrosion inhibition of *Inula viscosa* root (IVR) parts holds promising potential across various industries. Chemical compounds found in these roots have demonstrated corrosion inhibitory properties, offering opportunities for eco-friendly and efficient applications. Understanding these mechanisms could reduce maintenance costs and environmental impacts associated with metal corrosion.

## 2 Materials and methods

### 2.1 Preparation of MS

This study focused on material selection, with MS as the primary choice. MS is widely used in various industries due to its cost-effectiveness and good conductivity. While other elements can be present, carbon significantly influences the steel’s properties. Please refer to Table 1 for a detailed chemical breakdown of MS’s composition.

**Table 1:** Mass fraction of the chemical composition of the steel components used

Elements	Fe	Si	C	Mn	S	P	Al
Mass (%)	99.21	0.38	0.21	0.05	0.05	0.09	0.01

The MS specimens used in the test underwent mechanical polishing with sandpaper on various cores. Subsequently, impurities were removed from the sample by immersing it in distilled water, followed by degreasing with an analytical solution of acetone, and finally, they were dried before each trial. The choice of hydrochloric acid (HCl) is justified by its versatility, particularly its common use in the acid pickling of steel.

### 2.2 Electrolytic medium

In this study, the corrosive solution of interest is a HCl solution with a specific molarity. This solution is prepared by diluting HCl with distilled water, with the original HCl having a concentration of 37%. The resulting solution has a specific gravity of 1.19. The inhibitors used in this context are prepared with 1 M HCl and range in content from 0.25 to 1 g/L.

### 2.3 Sample collection and authentication

**Figure 1:**
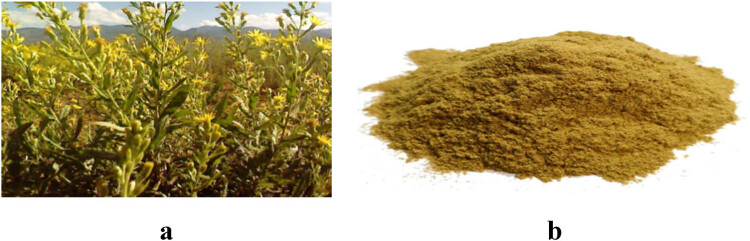
*I. viscosa* plant (a) and powder (b).

*I. viscosa* plants were gathered based on ethnopharmacological insights from communities near the Moulay Yaâcoub in Morocco (Figure 1). This collection was carried out in collaboration with local authorities and in full accordance with the United Nations Convention on Biodiversity. The invaluable assistance of a traditional healer played a crucial role throughout this process. Professor Amina Bari, a botanist at the Faculty of Science, Dhar Mahraz, Fes, confirmed the plant material’s identity. Furthermore, a reference specimen (**212As02 Iv 001**) [48] was officially archived in the herbarium of the Botany Department.

### 2.4 Inhibitor’s preparation

The extraction method is critical to ensure that the desired components are extracted from the complex natural matrix without damaging them. A wide range of techniques have been reported, and the results of this review have shown the use of both conventional and unconventional methods for extracting bioactive compounds. The commonly reported extraction methods were maceration and Soxhlet [49–51].

The roots of the *Inula viscosa* plant are dried and uniformly ground in a mortar to obtain a fine powder. In total, 200 g of *I. viscosa* plant root powder was extracted with 500 mL of water and placed in a container under continuous agitation for 72 h. The mixture was then filtered through a filter paper (Whatman No. 2, 125 mm) to separate the water-based solvent contained in the root extract. The remaining solvent was removed using a rotary evaporator at temperatures above 90°C under low pressure. After the solvent was evaporated using the rotary evaporator, the sample was transferred to an oven maintained at a constant temperature. This additional step allowed for the elimination of any residual water molecules, ensuring the purity of the extract. The final result, obtained after this drying step in the oven, is referred to as the IVR water extract, ready to be used in our study. The resulting aqueous extract was then stored at 4°C. Ethanol extracts were obtained by subjecting 150 g of root powder material from the *I. viscosa* plant to a Soxhlet extraction process for 6 h, using approximately 100 mL of solvent. Subsequently, the filtered extracts were evaporated under vacuum using a rotary evaporator at temperatures exceeding 40°C until completely dried. These concentrated extracts were then stored as residues at 4°C. Mixed extracts (70 mL of ethanol + 30 mL of water) were obtained by subjecting 200 g of root powder material from the *I. viscosa* plant to a Soxhlet extraction process for 6 h, using approximately 300 mL of solvent. Subsequently, the filtered extracts were evaporated under vacuum using a rotary evaporator at temperatures exceeding 40°C until completely dried. These concentrated extracts were then stored as residues at 4°C.

### 2.5 HPLC-DAD analysis

Extracts from the roots of the *I. viscosa* plant, including the ethanolic extract, aqueous extract, and mixed extract, were prepared at a concentration of 50 mg/mL, after being prepared, 0.45 μm microfilters were used to filter these extracts. Phenolic chemicals were identified and quantified using high-performance liquid chromatography (HPLC) in conjunction with a UV detector that operated between 210 and 400 nm in wavelength. Specifically, a reverse-phase C18 column (250 × 4 mm, 5 μm) was filled with 40 µL of sample injection. 20% B for 0–25 min, 100% B for 25–30 min, and 20% B for 30–35 min were the elution gradients that were used. For sample elution, mobile phases A (water/0.5% phosphoric acid) and B (methanol) were used at a flow rate of 1 mL/min. A constant temperature of 40°C was used for the separating procedure. The processes used for compound identification followed the guidelines provided by earlier research [52,53,54].

### 2.6 Electrochemical technique

The electrochemical evaluations were carried out using a Versa-STAT 4 potentiostat controlled by Versa-Studio software. A small glass cell with three distinct electrodes was utilized for this procedure. In this cell, the specimen under investigation (MS 1 cm²) acted as the working electrode, while the Ag/AgCl electrode was the reference electrode. Additionally, a counter electrode was created using platinum wire. The test sample, characterized by a rectangular shape with a surface area of 1 cm², was immersed in the sample preparation for 30 min to establish an open circuit potential (OCP).

The corrosion current density values were measured under two different conditions: without the presence of the inhibitor and with the inhibitor

Furthermore, graphs depicting the potentiodynamic polarization (PDP) curves were generated by altering the potential within the range of ±250 mV/Ag/AgCl, with a scanning rate of 1 mV/s. The study involving electrochemical impedance spectroscopy (EIS) was also carried out across a frequency range from 100 mHz to 100 kHz at the OCP, using an alternating waveform with an amplitude of 10 mV [55].

### 2.7 Surface analysis

The Scanning electron microscopy (SEM)-Energy-dispersive X-ray (EDX) spectroscopy was used to analyze the structure and arrangement of the surface of MS samples under two different conditions: the first condition without the inhibitor and the second condition with an inhibitor at a concentration of 1 g/L [52,56,57]. This examination was conducted using an environmental SEM. Specifically, the QUANTA 200 model, equipped with an EDX probe, operates at a 15 kV accelerating voltage. This technique allowed for the examination of the elemental composition of the MS surface and provided insights into its surface morphology and any changes that occurred in the presence of the inhibitors.

### 2.8 Quantum chemical calculations

The theoretical study is very interesting and useful in the corrosion inhibition field to correlate the reactivity of the obtained molecule structures with their inhibition performance. Therefore, Density Functional Theory (DFT) calculations were executed at B3LYP/6-311G (d, p) using Gaussian 09 and GaussView 5.0.8 software. The calculations were executed in water to approach the experimental conditions. Various global descriptors such as *E*_HOMO_, *E*_LUMO_, energy gap (Δ*E*_gap_), global hardness (*η*), global softness (*σ*), electronegativity (*χ*), and the fraction of electrons transferred (Δ*N*_110_) were extracted using the following equations, where *ΦFe/110* is 4.82 eV and *ηFe(110)* = 0 eV:

(1) \[\Delta {E}_{{\mathrm{gap}}}={E}_{{\mathrm{LUMO}}}-{E}_{{\mathrm{HOMO}}},]\]

(2) \[\chi =\frac{1}{2}({E}_{{\mathrm{HOMO}}}+{E}_{{\mathrm{LUMO}}}),]\]

(3) \[\eta =\frac{1}{2}({E}_{{\mathrm{LUMO}}}-{E}_{{\mathrm{HOMO}}}),]\]

(4) \[\sigma =\frac{1}{\eta },]\]

(5) \[\Delta {\varepsilon }_{{\mathrm{back}}-{\mathrm{donation}}}=\frac{-\eta }{4},]\]

(6) \[\Delta {N}_{{\mathrm{metal}}}=\frac{{\Phi }_{{\mathrm{metal}}}-{\chi }_{{\mathrm{inh}}}}{2({\eta }_{{\mathrm{metal}}}+{\eta }_{{\mathrm{inh}}})}=\frac{{\Phi }_{{\mathrm{metal}}}-{\chi }_{{\mathrm{inh}}}}{2{\eta }_{{\mathrm{inh}}}}.]\]

Monte Carlo (MC) simulation was carried out to gain insight into the adsorption approach of the tested molecular structures and their interactions with the iron surface. The simulated system considered the inhibitor molecules, 180 water molecules, the acidic solution (6H_3_O^+^ and 6Cl^−^ ions), and the steel surface composed of iron atoms. All system components under study were optimized using a COMPASS force field before performing the simulation using the Adsorption Locator module in Materials Studio 7.0 software. Additionally, the Fe (110) crystal was constructed with a 30 Å edge to provide sufficient depth and then enlarged to draw a supercell (10 × 10).

## 3 Results and discussion

### 3.1 HPLC analysis

**Figure 2:**
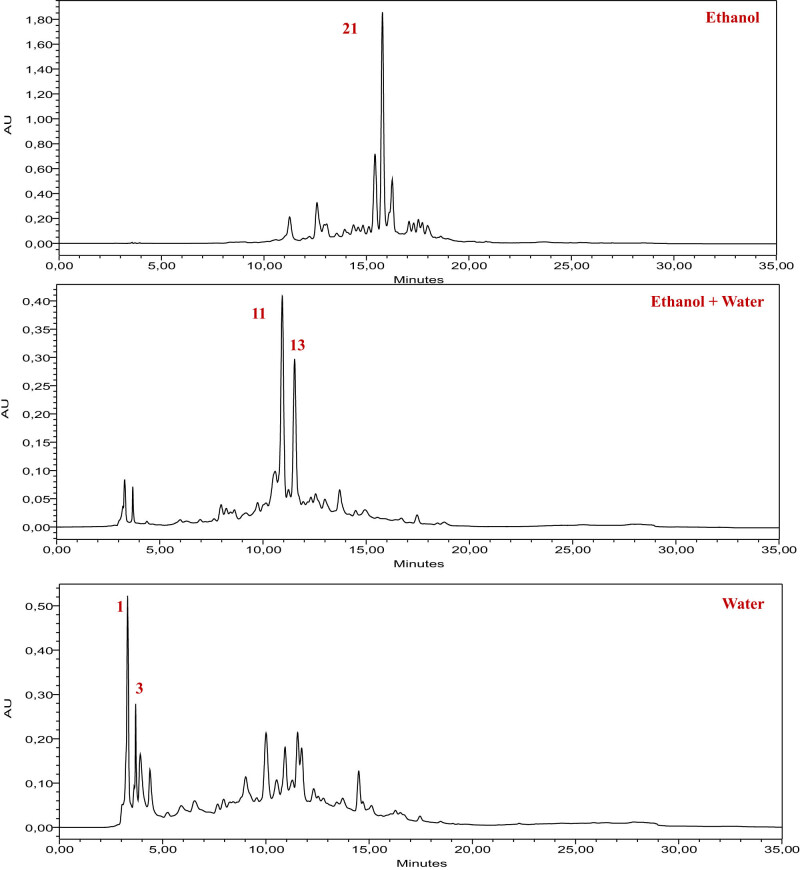
HPLC-DAD chromatogram of extracts from the root part of the *I. viscosa* plant, recorded at a wavelength of 320 nm, using the standards mentioned in the table above.

The analysis of extracts from the root part of the *I. viscosa* plant using HPLC revealed a high content of bioactive molecules, including flavonoids, polyphenols, and phenolic acids. The results of this analysis are presented in Table 2 and Figure 2, providing a detailed identification of compounds in the three studied extracts. The ethanolic extract of the root part highlighted the predominance of bioactive molecules such as catechin, vanillin, and quercetin 3-*O*-β-d-glucoside. The mixed extract (water + ethanol) showed a significant concentration of syringic acid, 3-hydroxybenzoic acid, and catechin. Finally, the aqueous extract exhibited an abundance of gallic acid, 4-hydroxybenzoic acid, and catechin. The analysis of the chromatograms indicates a diversity in the chemical composition of the various extracts while underscoring the presence of the same bioactive molecules in several of them, with varying concentrations depending on the solvent used. Previous studies have also confirmed the presence of compounds similar to those discovered in our research. Ten distinct phenolic compounds were identified in *I. viscosa* according to an investigation reviewed by Ozkan et al. These include gallic acid, caffeic acid, rutin, luteolin, kaempferol, rosmarinic acid, myricetin, quercetin, coumarin, and apigenin [58]. 21 different polyphenols are visible in the polyphenolic profile of the EtOAc extract of *I. viscosa*, which was determined through analysis. Five of these are flavonoids, namely, derivatives of quercetin, luteolin, naringin, and apigenin; the other four are phenolic acids, specifically caffeic acid, galloylquinic acid, and two isomers of di-O-caffeoylquinic acid [59]. Based on the information gathered from the analysis of phenolic compounds, it was discovered that *I. viscosa* plants have high levels of hyperoside, protocatechuic acid, and chloridric acid [60].

**Table 2:** Chromatographic analysis using HPLC of the compounds identified within the various extracts obtained from the roots of the *I. viscosa* plant

No.	Standards	Tr (min)	Ethanol extract	Mixed extract (ethanol + water)	Extract water
1	Gallic acid	3.26	ND	5.88	23.44
2	NI	3.69	ND	2.35	8.50
3	Catechin	3.92	ND	ND	10.84
4	Salicylic acid	4.38	ND	ND	5.85
5	NI	6.55	ND	ND	2.92
6	NI	7.97	ND	2.16	ND
7	Caffeic acid	9.02	ND	ND	2.74
8	NI	9.64	ND	1.31	ND
9	4-hydroxybenzoic acid	10.01	ND	ND	10.77
10	Catechin hydrate	10.56	ND	10.52	3.83
11	Syringic acid	10.93	ND	41.19	8.21
12	NI	11.27	6.79	ND	3.69
13	3-hydroxybenzoic acid	11.54	ND	27.32	8.79
14	Vanillic acid	11.73	ND	ND	6.72
15	Vanillin	12.57	6.84	2.36	ND
16	NI	12.94	5.72	2.46	ND
17	Naringin	13.00	3.01	4.44	ND
18	Cinnamic acid	14.44	2.81	ND	3.71
19	Ferulic acid	14.83	2.52	ND	ND
20	*p*-coumaric acid	15.24	16.54	ND	ND
21	Sinapic acid	15.43	29.97	ND	ND
22	Succinic acid	15.78	4.13	ND	ND
23	Quercetin 3-*O*-β-d-glucoside	16.11	10.55	ND	ND
24	Rutin	16.25	3.28	ND	ND
25	Quercetin	17.08	2.19	ND	ND
26	Kaempferol	17.307	2.77	ND	ND
27	Apigenin	17.532	2.88	ND	ND

ND: non detected; NI: not identified.

### 3.2 Electrochemical study

It is imperative to determine the period during which the electrode must remain immersed in the electrolyte to achieve the electrochemical equilibrium of the solution. Given that the corrosion potential is primarily determined by the stability of the working electrode in the electrolyte, it is crucial to establish the required duration for the corrosion potential to reach stabilization. This study estimated that an immersion period of 30 min is necessary to achieve a 99% stabilization of the corrosion potential when immersing an iron metal plate in a molar HCl solution.

#### 3.2.1 OCP monitoring

**Figure 3:**
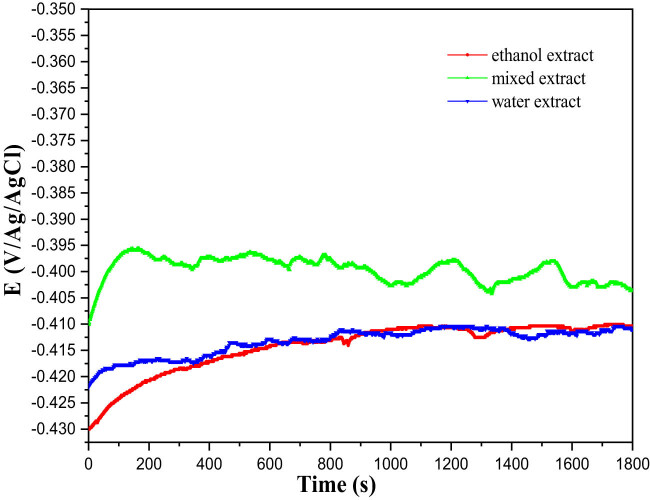
The progression of the OCP of MS in a 1 M HCl solution at 298 K, both with and without the presence of 1 g/L of the different inhibitor solutions under investigation.

This straightforward approach provides initial insights into the underlying mechanisms occurring at the metal and electrolyte interface [61]. Particular emphasis was placed on ensuring the OCP stability before initiating each polarization and impedance cycle. The change in OCP for the MS electrode is observed over time in the corrosive environment. This evolution is shown in Figure 3, both in the absence and presence of 1 g/L of *I. viscosa* plant extract at 298 K. In the presence of inhibitors, a slight shift towards more negative potential values is noticeable, indicating the primary impact of these compounds on the cathodic reactions. Additionally, 30 min is sufficient to reach the equilibrium potential.

#### 3.2.2 Polarization curves (steady-state electrochemical method)

**Figure 4:**
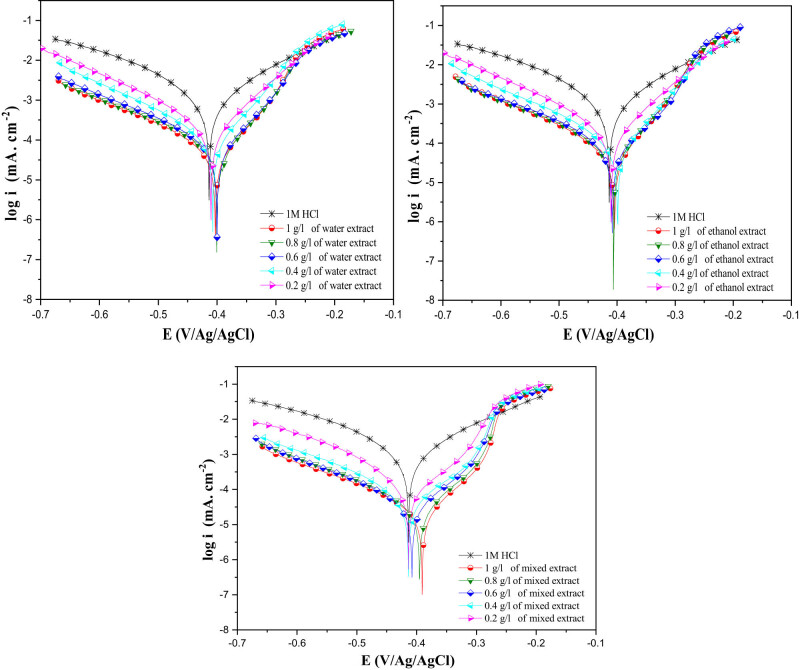
PDP curves for MS in 1 M HCl with and without different IVR concentrations at 298 K.

Electrochemical parameters such as cathodic slopes *ß*_c_ corrosion potential, corrosion current densities, etc., were carefully selected and organized in Table 3. Polarization curves depicting the behavior of MSs in an acidic environment, both without and with the various detected inhibitors, are shown in Figure 4. The corrosion inhibition efficiency values (IE%) were calculated using the following equation:

\[{\mathrm{IE}} \% =\frac{{i}_{{\mathrm{corrB}}}-{i}_{{\mathrm{corr}}/{\mathrm{inh}}}}{{i}_{{\mathrm{corrB}}}}\times 100.]\]

**Table 3:** Electrochemical parameters were derived from PDP curves for MS corrosion in a 1 M HCl solution at 298 K, with varying concentrations of IVR

	Conc.	−*E*_corr_	*i*_corr_	−*β*_c_	*η*_PDP_
	(g/L)	(mV/Ag/AgCl)	(µA cm^−2^)	(mV dec^−1^)	(%)
1 M HCl	—	498	983	140	—
Ethanol	1	405	51	128	94.8
	0.8	407	61	127	93.7
	0.6	409	74	126	92.4
	0.4	400	103	133	89.5
	0.2	410	212	134	78.4
Mixed (water + ethanol)	1	390	22	128	97.7
	0.8	397	31	130	96.8
	0.6	409	37	131	96.2
	0.4	413	63	129	93.5
	0.2	414	155	118	84.2
	1	404	46	126	95.3
Water	0.8	401	57	134	94.2
	0.6	402	67	135	93.1
	0.4	409	121	136	87.6
	0.2	411	215	135	78.1

Stationary electrochemical techniques were employed to assess the effectiveness of *I. viscosa* plant extracts obtained using water, a mixture of ethanol and water, and ethanol alone. These techniques mainly involve the analysis of curves describing the current/voltage relationships under potentiodynamic conditions at anodic and cathodic potentials.

Figure 4 illustrates the polarization curves for cathodic and anodic reactions in a 1 M HCl solution containing MS. Measurements were conducted at 298 K, including scenarios with and without the tested inhibitors at different concentrations, ranging from 0.25 to 1 g/L, at 25°C. The observed balancing in all samples can be attributed to the formation of an iron oxide/hydroxide layer on the surface of MS in the absence of inhibiting substances. However, in a 1 M HCl solution with an inhibitor, this stabilization results from the adsorption of inhibitor molecules on the MS surface. Table 3 illustrates the evolution of the corrosion rate of MS in HCl solutions with and without the inhibitor. The results indicate that current density (*i*_corr_) decreases progressively with increasing IVR inhibitor concentrations in the different extracts studied [62]. For instance, in the IVR mixed extract, when the inhibitor concentration is 0.2 g/L, the current density is 155 µA cm^**−**2^, but after an increase in the inhibitor concentration to 1 g/L, there is a decrease in current density of almost 22 µA cm^**−**2^. Using the PDP technique, the inhibitory power increases with the concentration, reaching 97.7% with the mixed extract and 94.8% with the ethanolic extract, and 95.3% with the water extract when the inhibitor concentration is 1 g/L.

The presence of the inhibitor influences both anodic and cathodic current densities. Cathodic curves exhibit similarities among all samples, indicating an unchanged cathodic reduction mechanism and hydrogen reduction controlled by pure activation kinetics. Furthermore, anodic curves are affected by the presence of the IVR extract in an acidic environment, altering the iron dissolution mechanism and forming a new protective layer. Electrochemical parameters were determined through polarization curve analysis, including cathodic slope (*β*_c_), corrosion potential (*E*_corr_), inhibition efficiency (*η*_PDP_), and corrosion current density (*i*_corr_), as summarized in Table 3. It is important to emphasize that the shift in the corrosion potential (*E*_corr_) is less than 85 mV, indicating that the IVR extract mitigates the corrosive effects of HCl through a mixed-type mechanism, as the *E*_corr_ differences fall within the ±85 mV range [63–67].

#### 3.2.3 EIS

The appearance of offensive chloride ions in the solution increases the speed of degradation of the MS surface by increasing the disintegration of the anode component, as described by the reactions involving Cl^−^ and Fe. To gain a deeper understanding of the kinetic behavior at the metal/solution interface, we notice two different cases. When the inhibitor does not exist, the impedance spectrum appears as a small half-circle, with a time constant observed in the Bode diagram. This result confirms the rapid decomposition of iron as well as the release of hydrogen. Conversely, in the second case, a high-frequency capacitive loop was evident when various IVR extracts were added to the 1 M HCl solution. Furthermore, increasing the concentration of different IVR extracts resulted in the enlargement of the semicircle. This observation suggests that the inhibitor influences the charge transfer mechanism between the metal and the solution and the adsorption of inhibitor molecules.

The specific phenomenon observed on the surface of MS in the impedance diagram of solid-state electrodes is known as frequency dispersion. This phenomenon can be attributed to several factors, including surface heterogeneity [68], the presence of impurities or dislocations [69], surface fragility, dispersion of active centers, and the formation of porous protective layers [70]. In our study of Bode diagram, we observed that in the absence of inhibitor, the impedance amplitude may be lower, indicating more active corrosion and less effective protection of the metal surface. Additionally, the peak of impedance amplitude may be located at a different frequency compared to the system with inhibitor, reflecting differences in ongoing electrochemical processes.

Conversely, after the addition of inhibitor at different concentrations, we observed an increase in impedance amplitude at certain frequencies, indicating a reduction in corrosion rate and improved protection of the metal surface. Furthermore, the peak of impedance amplitude may shift to lower or higher frequencies with the addition of inhibitor, suggesting a change in the dominant corrosion mechanism or in the adsorption processes of the inhibitor onto the metal surface.

**Figure 5:**
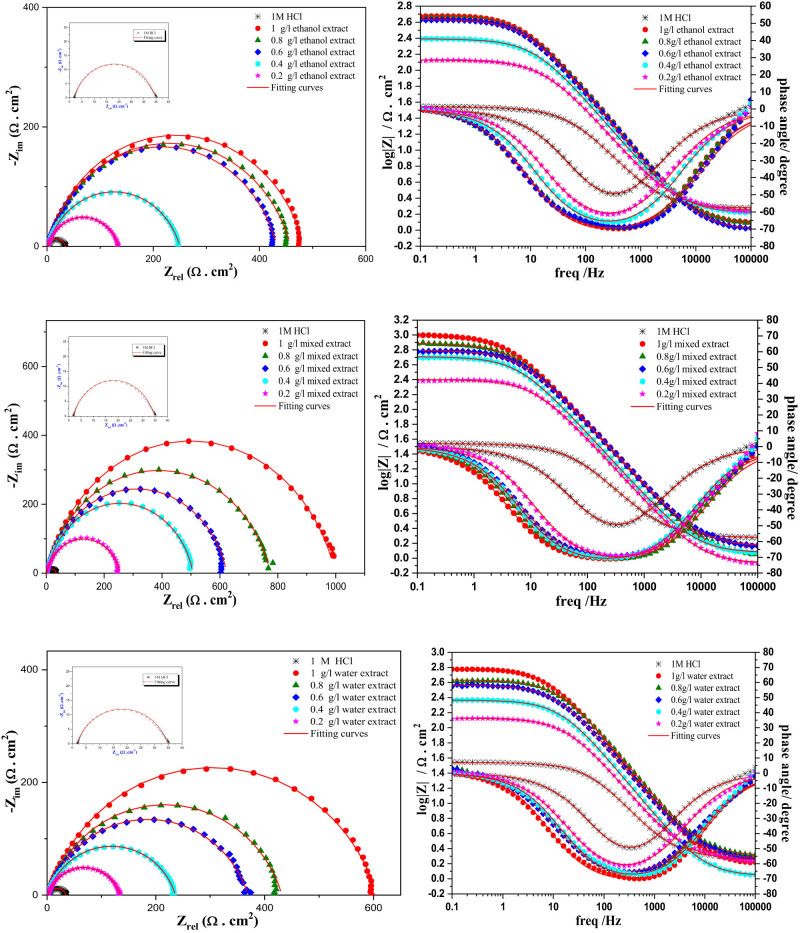
Nyquist and Bode diagrams for inhibitors extracted from the roots of *I. viscosa* plants at 298 K at different concentrations.

In the context of this study, the circuit model shown in Figure 5, which includes elements like solution resistance (*R*_s_), polarization resistance (*R*_p_), and double layer capacitance (*C*_dl_), is insufficient for accurately calculating the parameters of EIS. This model is designed for homogeneous systems. A more appropriate model should be employed to account for the observed frequency dispersion, including a frequency dispersion element like the constant phase element (CPE). The CPE parameter is calculated using an appropriate equation (equation (7)) [71,72].

(7) \[{Z}_{{\mathrm{cpe}}}\left={Q}^{-1}({i\omega )}^{-n},]\]

where *Q* represents the amplitude of the CPE, *i* represents the imaginary number of the CPE, *ω* represents the rotating frequency, and *n* represents the experimental coefficient.

**Figure 6:**
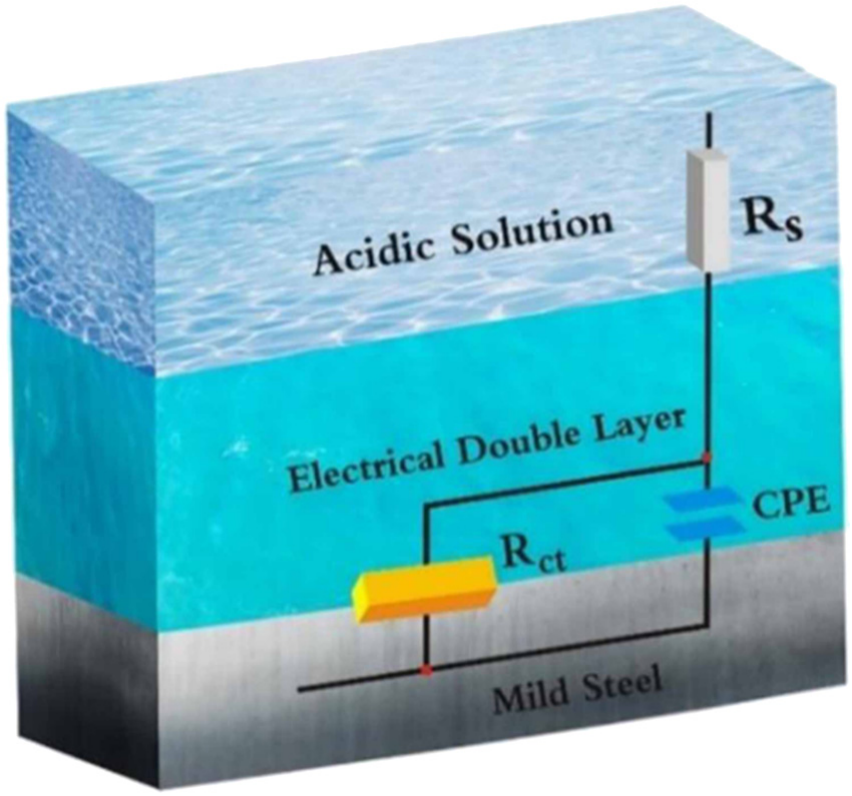
Equivalent circuit used to fit electrochemical data.

Electrolytic data, such as solution resistance (*R*_s_), charge transfer resistance (*R*_ct_), CPE and double layer capacitance (*C*_dl_), are presented in Table 4 (Figure 6).

(8) \[\eta {\mathrm{EIS}}{\boldsymbol{ \% }}=\frac{{R}_{{\mathrm{ct}}({\mathrm{inh}})}-{R}_{{\mathrm{ct}}}}{{R}_{{\mathrm{ct}}({\mathrm{inh}})}}\times 100,]\]

where *R*_ct(inh)_ is the inhibitor charge transfer resistance and *R*_ct_ is the charge transfer resistance of 1 M HCl.

**Table 4:** EIS values for MS in corrosive media 1 M HCl without and with inhibitor IVR extracts

	Conc.	*R*_s_	*R*_ct_	*C*_dl_	*n*_dl_	*Q*	*Ɵ*	*ƞ*_imp_
	(g/L)	(Ω cm^2^)	(Ω cm^2^)	(µF cm^−2^)				(%)
1 M HCl	—	1.76	33.2	89.10	0.784	312.7	—	—
Ethanol	1	1.20	479.1	43.03	0.837	80.75	0.930	**93.0**
	0.8	1.18	453.3	44.46	0.829	86.41	0.926	92.6
	0.6	0.99	431.5	46.78	0.827	91.58	0.923	92.3
	0.4	1.63	245.7	52.78	0.823	113.7	0.864	86.4
	0.2	1.76	132.9	64.54	0.806	162.0	0.750	75.0
Mixed (water + ethanol)	1	1.44	996.7	40.96	0.836	69.22	0.966	**96.6**
	0.8	1.10	772.9	41.08	0.840	71.35	0.957	95.7
	0.6	1.44	631.4	42.49	0.846	74.18	0.947	94.7
	0.4	1.18	511.1	51.26	0.844	90.47	0.935	93.5
	0.2	0.85	254.4	54.08	0.843	105.40	0.869	86.9
Water	1	1.56	595.3	29.83	0.816	62.64	0.944	**94.4**
	0.8	2.01	429.7	30.16	0.817	66.66	0.922	92.2
	0.6	1.82	367.8	38.34	0.805	87.93	0.909	90.9
	0.4	1.08	232.1	48.72	0.825	106.7	0.856	85.6
	0.2	1.75	133.1	64.59	0.806	162.0	0.678	75.0

Bold values show maximum percentages of corrosion inhibition.

Solution resistance (*R*_s_) is an important measure in electrolytic systems as it represents the resistance to electrical conduction offered by the solution itself. This value is essential for assessing the electrical conductivity of the medium and understanding how ions move through the solution. A high *R*_s_ may indicate low solution conductivity, which can influence ongoing electrochemical processes. For instance, in the context of corrosion inhibitor studies, a higher *R*_s_ may suggest reduced ion mobility in the solution, which can impact the effectiveness of corrosion inhibition. In summary, the *R*_s_ value in Table 4, ranging from a minimum of 0.85 to a maximum of 2.01, provides valuable insight into the electrical conductivity of the system and its involvement in the studied electrochemical processes.

According to the data presented in Table 4, it can be observed that the values of ndl are significantly higher than those of the control sample (blank). This observation suggests that the presence of inhibitor in the chloridric acid solution reduces the irregularity of the MS surface [73]. This reduction is probably attributed to the formation of an organic film on the steel surface. Additionally, the phase shift values (*n*) when inhibitors are introduced are very close to unity. This observation implies that the electric double layer formed by the adsorption of IVR bioactive molecules at the metal-electrolyte interfaces behaves like a pseudo-capacitor. The most evident observation lies in the low values of solution resistance, approximately 1–2 Ω cm^2^, and charge transfer resistance, around 33.2 Ω cm^2^, observed in a corrosive environment, confirming the rapidity of the corrosion processes [74]. In comparison, significantly higher values of solution resistance and charge transfer resistance have been recorded, confirming that the addition of corrosion inhibitors increased resistance against corrosion processes [75].

In our study, it is noticeable that the *R*_ct_ (charge transfer resistance) value for the blank (absence of inhibitor) is 33.2 Ω cm². Conversely, after the addition of inhibitors, a remarkable increase in *R*_ct_ is observed, ranging from a maximum of 996.7 Ω cm^2^ to a minimum of 132.9 Ω cm^2^. This variation in *R*_ct_ is linked to the variation in IVR extract and the concentration of each IVR extract. While the values of the double-layer capacitance (*C*_dl_) decrease, in our case, in the absence of inhibitor, the value of *C*_dl_ is 89.10 µF cm^−2^. After the addition of inhibitor, a rapid decrease in double-layer capacitance is observed, reaching a maximum value of 64.59 µF cm^−2^ and a minimum value of 29.83 µF cm^−2^. This phenomenon leads to a reduction in the rate of charge and discharge at the interface between the working electrode and the solution. The roughness of the MS surface and the corrosive environment of the CPE contribute systematically to the formation of a double layer [76].

The EIS parameters obtained confirmed that the corrosion inhibition efficiency was higher with various IVR extracts and different concentrations, reaching maximum values of up to 96.6% in the mixed extract (ethanol + water). This can be explained by solvent interaction: Water and ethanol are polar solvents, meaning they have the ability to interact with both polar and non-polar molecules. This interaction can facilitate the dissolution and dispersion of inhibitor molecules, leading to better coverage and adherence to the metal surface, thus improving inhibition performance. Additionally, synergistic effects may also be at play: The combination of water and ethanol can create synergistic effects that enhance inhibition performance beyond what would be achieved with either solvent alone. These synergistic effects could result from changes in the composition of the mixture, leading to enhanced interactions between the components [77].

#### 3.2.4 Isotherm adsorption

In order to ascertain accessible information regarding the adsorption capabilities of inhibitors derived from the roots of the *I. viscosa* plant, the outcomes of experimental EIS data were assessed through parallel lines corresponding to diverse adsorption isotherms (Langmuir, Temkin, Frumkin, Freundlich, Flory-Huggin, and El-Awady), employing linear equations (Table 5) [78].

**Table 5:** Linear equations for isotherms

Isotherms	Linear equations	Descriptions
Langmuir	\[\frac{{{\boldsymbol{C}}}_{\text{inh}}}{{\boldsymbol{\theta }}}=\hspace{1em}\frac{{\bf{1}}}{{\boldsymbol{K}}}+{C}_{\text{inh}}]\] (9)	*K*: Adsorption coefficient, *C*_inh_: Inhibitor concentration, *ϴ*: Inhibitor retention rate.
El-Awady	\[\mathrm{ln}\left(\phantom{\rule[-0.75em]{}{0ex}},\frac{\theta }{1‒\theta }\right)=y\mathrm{ln}K+y\mathrm{ln}{C}_{{\mathrm{inh}}}]\] (10)	1/*y* represents the quantity of water molecules removed by a single molecule of the inhibitor compound.
Flory-Huggins	\[\mathrm{ln}\left(\phantom{\rule[-0.75em]{}{0ex}},\frac{\theta }{{C}_{{\mathrm{inh}}}}\right)=\mathrm{ln}K+x\mathrm{ln}(1‒\theta )]\] (11)	*X* represents the number of adsorbed H_2_O molecules substituted by inhibitor compounds.
Freundlich	\[\mathrm{ln}\text{}\theta \hspace{0.25em}=\text{}\mathrm{ln}\text{}{K}+\text{}Z\text{}\mathrm{ln}{C}_{\text{inh}}]\] (12)	*Z*, where 0 < *Z* < 1, indicates that the adsorption of the inhibitor on the metal surface is facile
		When *Z* = 1, it denotes a moderate adsorption of the inhibitor on the metal surface
		If *Z* > 1, it implies a challenging adsorption behavior of the inhibitor
Frumkin	\[\mathrm{ln}\left(\phantom{\rule[-0.75em]{}{0ex}},\frac{{\boldsymbol{\theta }}}{(1- \theta){{\boldsymbol{C}}}_{\text{inh}}}\right)=\text{}\mathrm{ln}\text{}K\text{}+\text{}2a\theta ]\] (13)	*a* represents the interaction factors between adsorbed molecules, which can involve either repulsive or attractive forces
Temkin	\[\theta \text{}\left=\text{}\frac{\text{‒}1}{2a}\mathrm{ln}\left(K\left)\text{‒}\frac{1}{2a}\mathrm{ln}\left({C}_{\text{inh}})]\] (14)	*a* represents the coefficient for the interaction, whether it is repulsion or attraction, among adsorbed compounds

**Figure 7:**
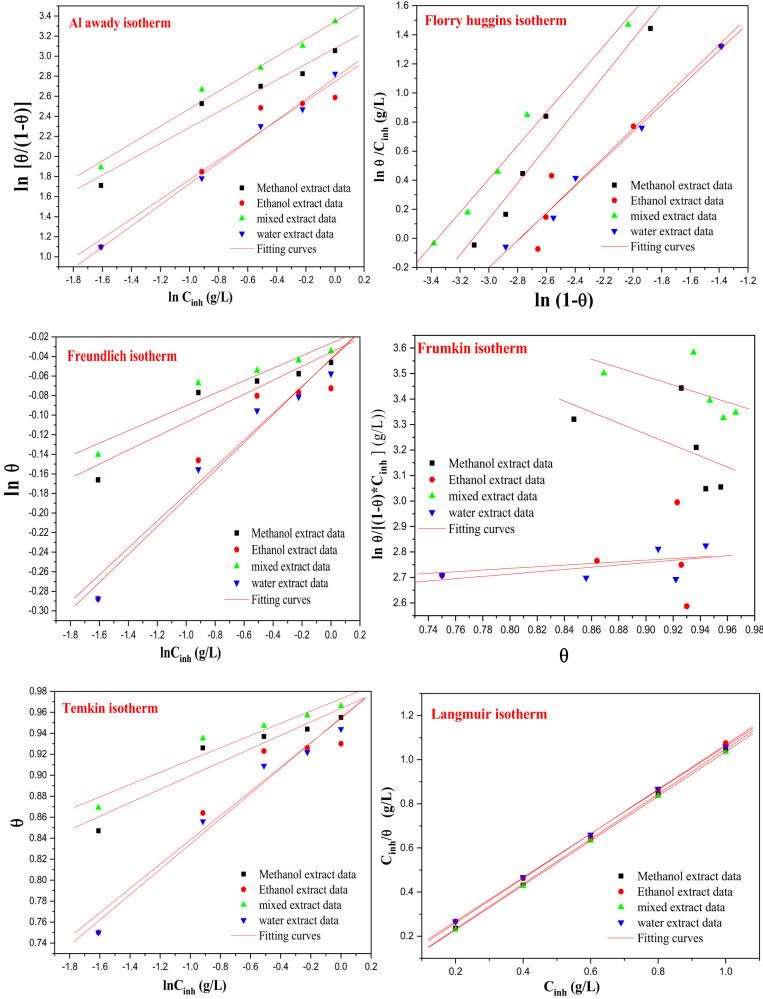
Different isothermal models for MS in a pickling medium containing different concentrations of IVR extract at 298 K.

The adsorption process of various extracts from the roots of the *I. viscosa* plant was examined using different isothermal models. From the data presented in Table 6 and Figure 7, it is observed that the correlation coefficient values were close to 1 in the Langmuir model, except for the Frumkin model, which showed moderate correlation coefficients varying with solvent variation (min 0.16–max 0.59). Hence, given the adsorption constants, it can be suggested that the adsorption phenomenon of the studied inhibitors can be better represented by the Langmuir model for the different extracts studied. It is noteworthy that the Langmuir model exhibits a very high correlation coefficient of 0.99 and a slope near to unity. The Freundlich model is excluded due to its very low *K* values, despite having a high correlation coefficient, with the adsorption values of the studied inhibitors being far from the fitting. In the El-Awady model, a very strong correlation coefficient is observed along with \[\Delta{G}_{\text{ads}}^{0}]\] values close to 25 kJ/mol, indicating the formation of physisorption bonds. Conversely, the Frumkin model is excluded due to its weak correlation coefficients with very low \[\Delta{G}_{\text{ads}}^{0}]\] values. The Langmuir model shows an almost perfect correlation with \[\Delta{G}_{\text{ads}}^{0}]\] values close to 25 kJ/mol, and all values of the studied inhibitors are well placed in the fitting curve, allowing us to conclude that the adsorption phenomenon of the studied inhibitors can be better represented by the Langmuir model for the different extracts studied. Furthermore, based on the Temkin isotherm, the negative values of the (a) parameter indicate the presence of an attractive interaction at the interface between MS and inhibitors extracted from the roots of the *I. viscosa* plant. This hypothesis is also supported by the Frumkin isotherm, especially in the case of ethanol and aqueous extracts [79].

**Table 6:** Key parameters were obtained by testing different isothermal models

Isotherms	Inhibitors	*R*²	Parameters		*K*	\[\Delta{G}_{\text{ads}}^{0}]\] (kJ/mol)
Freundlich	Ethanol	0.95	*z*	7.26	0.95	−17.02
	Mixed	0.96		15.66	0.97	−17.06
	Water	0.98		7.00	0.95	−17.02
El-Awady			1/*y*			
	Ethanol	0.97		1.02	16.6	−24.09
	Mixed	0.99		1.15	47.1	−26.67
	Water	0.99		0.94	16.2	−24.02
Frumkin			*a*			
	Ethanol	0.16		−0.16	8.40 × 10^−2^	−10.98
	Mixed	0.59		0.84	6.69 × 10^−3^	−4.71
	Water	0.53		−0.22	9.50 × 10^−2^	−11.29
Florry-Huggins			*x*			
	Ethanol	0.96		0.96	14.6	−23.77
	Mixed	0.99		1.14	46.8	−26.66
	Water	0.99		0.93	13.4	−23.56
Temkin	Methanol		*a*			
	Ethanol	0.95		−4.31	3.78 × 10^3^	−37.55
	Mixed	0.96		−8.54	1.66 × 10^7^	−58.34
	Water	0.98		−4.14	2.76 × 10^3^	−36.77
Langmuir			Slope			
	Ethanol	0.99		1.009	17.1	−24.17
	Mixed	0.99		1.009	36.9	−26.07
	Water	0.99		0.992	14.6	−23.77

### 3.3 Surface morphological studies

**Figure 8:**
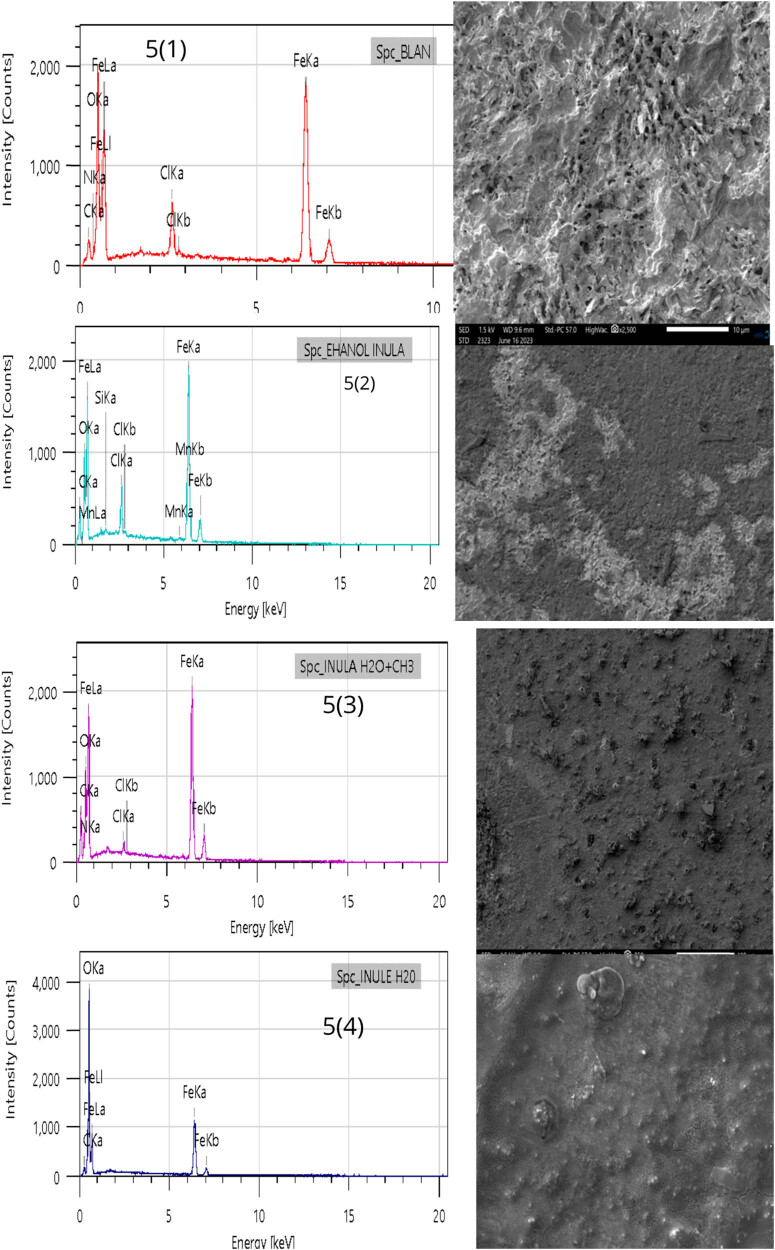
SEM/EDX images of MS in a molar HCl solution under investigation, both without and with the presence of inhibitors, following 6 h of immersion.

Figure 8 illustrates the morphological attributes of MS after a 6-h immersion in 1 M HCl, both in the absence and presence of a 1 g/L concentration of various root extracts from the *I. viscosa* plant. In Figure 8(1), the topography of the sample surface is corroded after immersion in 1 M HCl at 298 K for 6 h, resulting in irregular surfaces due to the effects of corrosion. Conversely, samples immersed in 1 M HCl containing concentrations of 1 g/L ethanol, mixed extract, and aqueous inhibitors of the root of the *I. viscosa* plant (Figure 8(2)–(4)) show smoother surfaces. This smoothness implies that the primary inhibitors are absorbed by the metal surface, generating a protective layer that shields the metal from the corrosive environment, thus preserving it from the damaging effects of corrosion [80].

Table 7 presents the weight proportions of various elements obtained through EDX analysis of the steel surface in a 1 M HCl solution. These measurements were conducted at a temperature of 298 K, both in the presence and absence of inhibitors at a concentration of 1 g/L. The experimental EDX spectrum of the MS sample reveals characteristic peaks for its fundamental components, including silicon (Si), aluminum (Al), carbon (C), manganese (Mn), chromium (Cr), and iron. When the sample is immersed in the uninhibited solution, the mass percentages for MS indicate a significant presence of chloride (Cl) and oxygen (O) elements. These elements are attributed to the formation of corrosion products on the steel surface. However, with the introduction of inhibitors, these rates decrease, thus demonstrating the protective effectiveness of these antioxidants on the steel surface against corrosive attacks. They contribute to reducing chloride ion penetration and preventing iron oxidation at the metal surface [49].

**Table 7:** Mass percentage of various constituents obtained from the EDX spectroscopy analysis of the steel surface was determined in a 1 M HCl solution at a temperature of 298 K, both in the presence and absence of inhibitors at a concentration of 1 g/L

Element	wt% of MS with HCl	wt% of MS with ethanol inhibitors	wt% of weight MS with mixed inhibitors	wt% of weight MS with water inhibitors
C	4.74 ± 0.06	7.70 ± 0.07	8.57 ± 0.07	3.73 ± 0.05
N	—	—	—	—
O	13.75 ± 0.11	6.80 ± 0.08	6.99 ± 0.08	5.98 ± 0.17
Cl	3.96 ± 0.06	1.2 ± 0.06	0.90 ± 0.04	0.82 ± 0.04
Fe	77.55 ± 0.51	80.54 ± 0.51	83.55 ± 0.51	65.14 ± 0.53
Mn	—	0.74 ± 0.08	—	—
Si	—	0.25 ± 0.03	—	—

### 3.4 Result of theoretical methods

**Figure 9:**
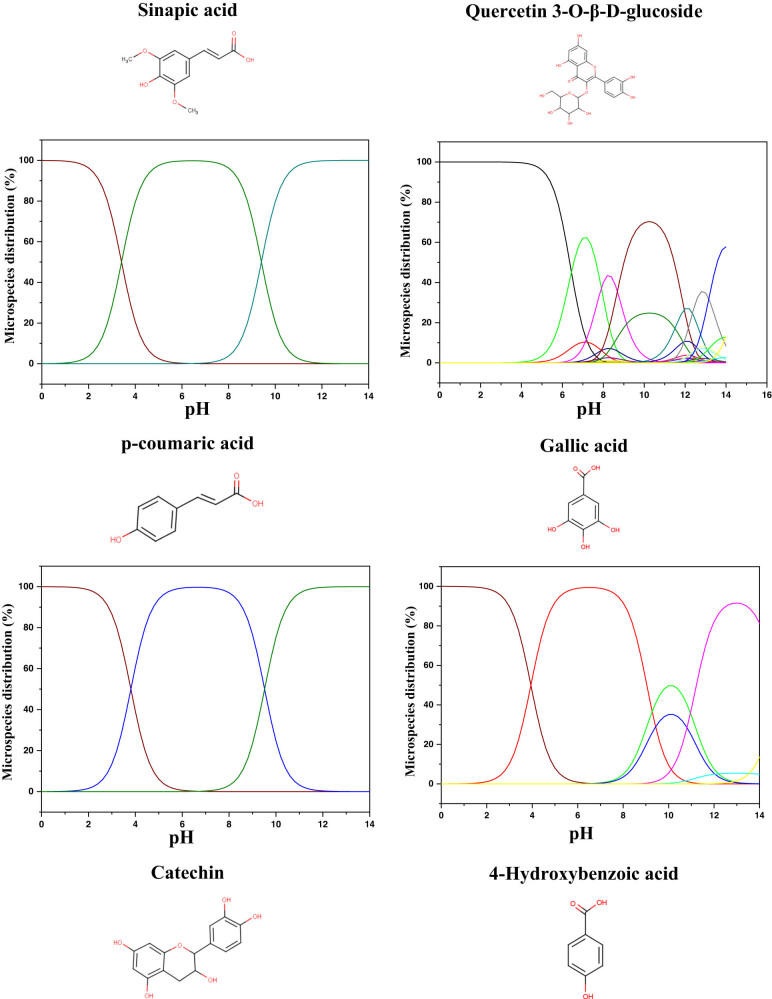

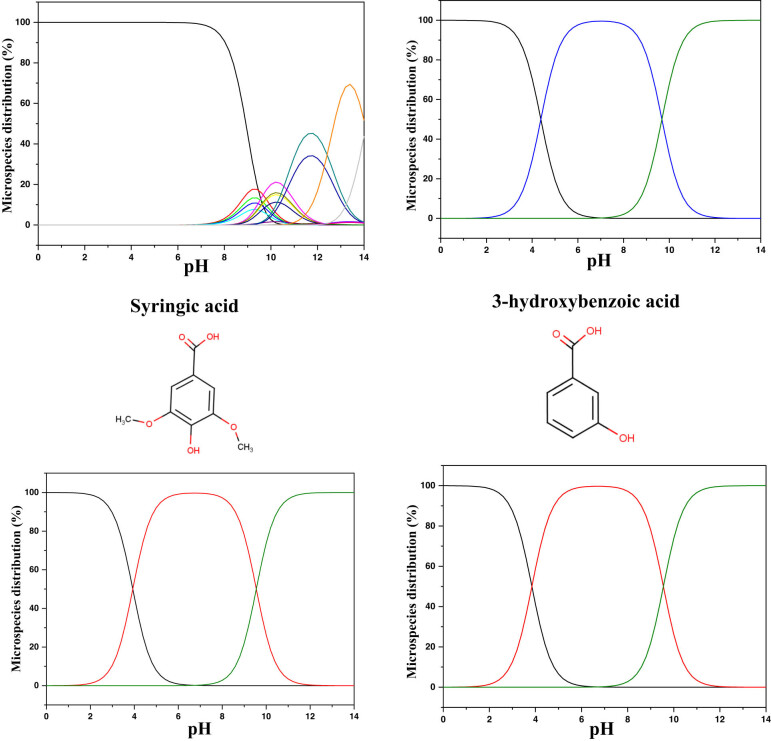
Microspecies distribution vs pH.

The quantum calculations help us understand and classify the reactivity of the molecules used as corrosion inhibitors and their interaction with the MS surface. Therefore, before the calculations, the predicted protonated form of the studied molecules was tested using MarvinSketch software [81,82]. The species distribution vs pH axes is presented in Figure 9. The theoretical calculations were conducted in the neutral state in the aqueous phase, and their descriptors were extracted and presented in Table 8. From the protonation results, it can be observed that all the molecules showed a high distribution of the neutral form in the acidic zone. Therefore, it can be suggested that these neutral forms are the predicted forms that can exist in the studied acidic solution (1 M HCl).

**Table 8:** Descriptors of reactivity for the neutral form of the studied molecules in aqueous phase

	Descriptors	*E*_HOMO_ (eV)	*E*_LUMO_ (eV)	Δ*E*_gap_ (eV)	*Ƞ* (eV)	*σ* (eV^−1^)	*χ* (eV)	Δ*E*_back-donation_	Δ*N*_Fe/110_
Ethanol	Sinapic acid	−5.9855	−2.0335	3.9519	1.9759	0.5060	4.0095	−0.4939	0.2050
	Quercetin 3-*O*-β-d-glucoside	−5.8726	−1.8335	4.0390	2.0195	0.4951	3.8530	−0.5048	0.2393
	*p*-coumaric acid	−6.2266	−1.9965	4.2301	2.1150	0.4728	4.1115	−0.5287	0.1674
Water	Gallic acid	−6.2824	−1.4305	4.8518	2.4259	0.4122	3.8564	−0.6064	0.1985
	4-Hydroxybenzoic acid	−6.6778	−1.3986	5.2791	2.6395	0.3788	4.0382	−0.6598	0.1480
	Catechin	−6.0320	−0.4378	5.5942	2.7971	0.3575	3.2349	−0.6992	0.2833
Water + ethanol	Syringic acid	−6.2843	−1.4523	4.8320	2.4160	0.4139	3.8683	−0.6040	0.1969
	3-Hydroxybenzoic acid	−6.5861	−1.6572	4.9289	2.4644	0.4057	4.1216	−0.6161	0.1416
	Catechin	−6.0320	−0.4378	5.5942	2.7971	0.3575	3.2349	−0.6992	0.2833

#### 3.4.1 DFT calculations

**Figure 10:**
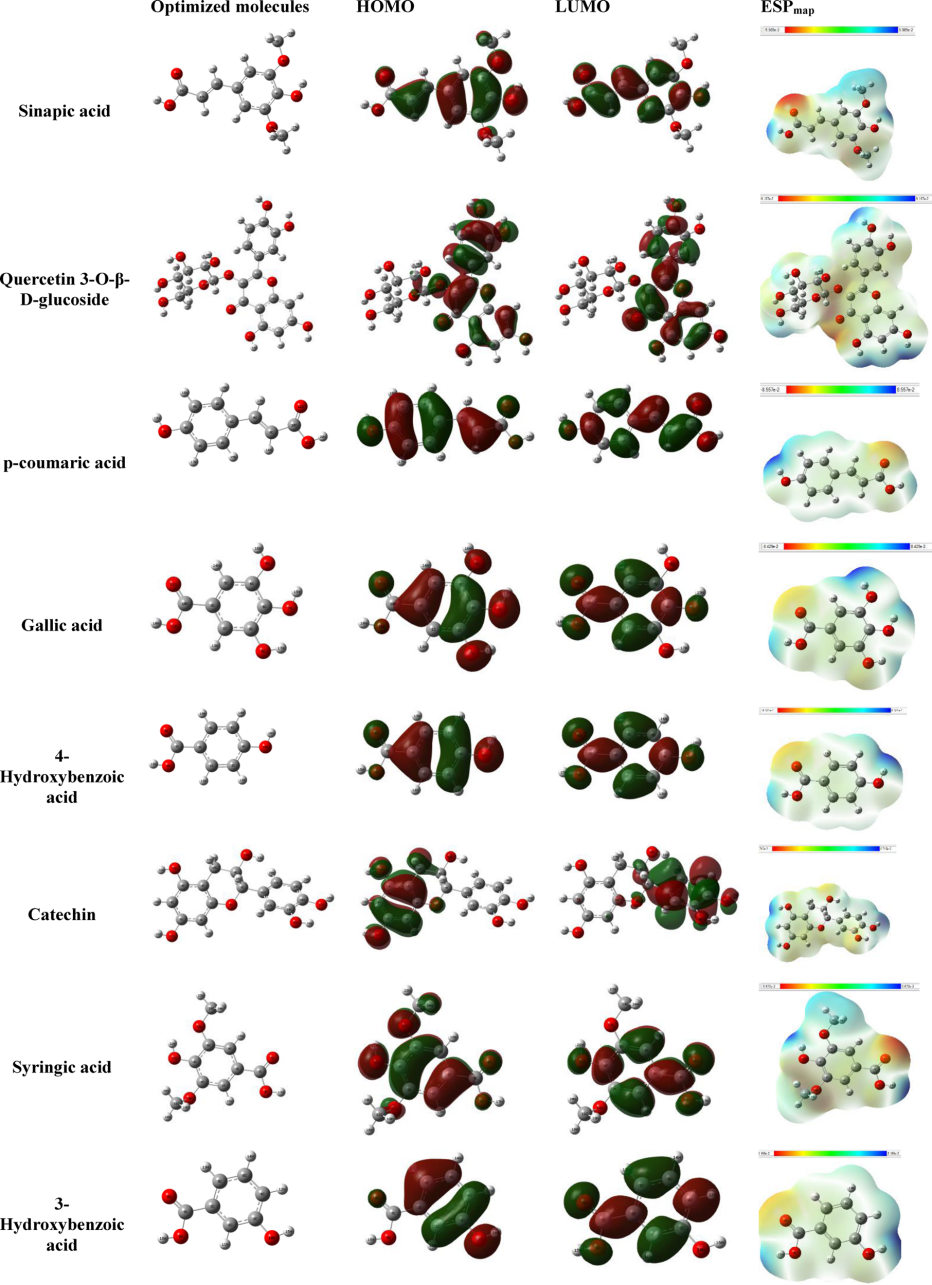
Optimized structure, LUMO, HOMO densities, and ESP map of detected molecules.

DFT plays a crucial role in understanding the inhibitory efficiency of the molecules found in the extract when adsorbed onto the MS surface. The adsorption process relies on donor-acceptor correlations governed by the electronic organization of the molecules. The electron-dense regions of these extract molecules can potentially transport their charges to the metal-liquid contact zone, especially towards the unsaturated orbitals of iron atoms. Figure 10 illustrates the electronic structure of the extracted molecules, highlighting the distribution of the Highest Occupied Molecular Orbital (HOMO) and Lowest Unoccupied Molecular Orbital (LUMO) for various molecules from the IVR extract. The graphs reveal that the HOMO orbital is primarily localized on the benzene function, methoxy function, and alcohol function for molecules such as sinapic acid, quercetin 3-*O*-β-d-glucoside, *p*-coumaric acid, gallic acid, 4-hydroxybenzoic acid, catechin, syringic acid, and 3-hydroxybenzoic acid. Simultaneously, the HOMO orbital is distributed across the entire surface of the molecule. Therefore, it can be concluded that the HOMO orbital contains π electrons from benzene functions and non-bonding electron pairs from heteroatoms, making them available for interaction with metal orbitals at the interface.

In addition, LUMO orbital is graphically located on the benzene function and the oxygen/carbon atoms. Consequently, the molecules of the extracts can be adsorbed on the surface of iron by accepting the electrons located in the orbitals occupied by the iron atoms [65].

Table 8 lists data regarding the fundamental characteristics of neutral forms within the predominant structure found in each IVR extract. These parameters encompass the estimation of HOMO, the quantum levels of LUMO, dipole moment values, and the energy gap (Δ*E*_gap_), which serve as indicators of the anticorrosion potential of the extracted molecules [66].

The lowest Δ*E*_gap_ values for IVR molecules (ethanolic extract) are as follows: 3.9519 eV for sinapic acid, 4.0390 eV for quercetin 3-*O*-β-d-glucoside, and 4.2301 eV for *p*-coumaric acid. On the other hand, the minimal Δ*E*_gap_ values for IVR molecules (aqueous extract) are 4.8518 eV for gallic acid, 5.2791 eV for 4-hydroxybenzoic acid, and 5.5942 eV for catechin. As for the minimal Δ*E*_gap_ values for molecules in the mixed IVR extract, they are, respectively, 4.8320 eV for syringic acid, 4.9289 eV for 3-hydroxybenzoic acid, and 5.5942 eV for catechin. These results suggest that the majority of molecules present in each extract exhibit remarkable anticorrosion reactivity.

Based on the chemical hardness/softness descriptors, it can be observed that Sinapic acid molecule has a high reactivity compared to the other components in the ethanolic extract. For the aqueous extract, the Gallic acid molecule showed a small hardness value and a high softness value compared to the other components that mostly existed in this extract, verifying the high reactivity of Gallic acid molecule. In addition, syringic acid molecule is the high reactive molecule in the mixed IVR extract. This observation leads us to suppose that the studied molecule can offer electrons to the metal surface, facilizing their adsorption onto the metal surface. Moreover, all the studied molecules showed a negative sign in Δ*E*_back-donation_ parameter, indicating the adsorption of these particles into the steel surface, and offering electrons to the d vacuum orbital of iron atoms. Furthermore, the electronegativity values results are gone with the same trend as the other parameters, confirming the reactivity order of the studied components.

The number of transferred electrons (Δ*N*_Fe/110_) can be considered as an indicator of the capacity to donate electrons. Referring to Table 8, it is evident that the Δ*N*_Fe/110_ values are positive, indicating that these molecules act as electron donors.

#### 3.4.2 MC simulation

**Figure 11:**
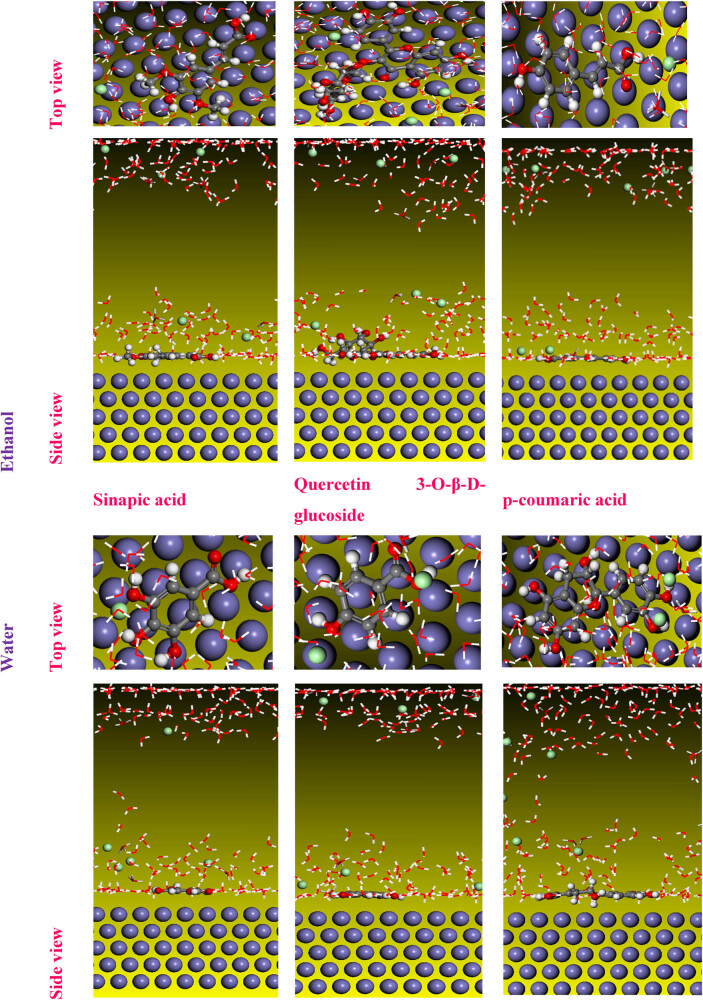

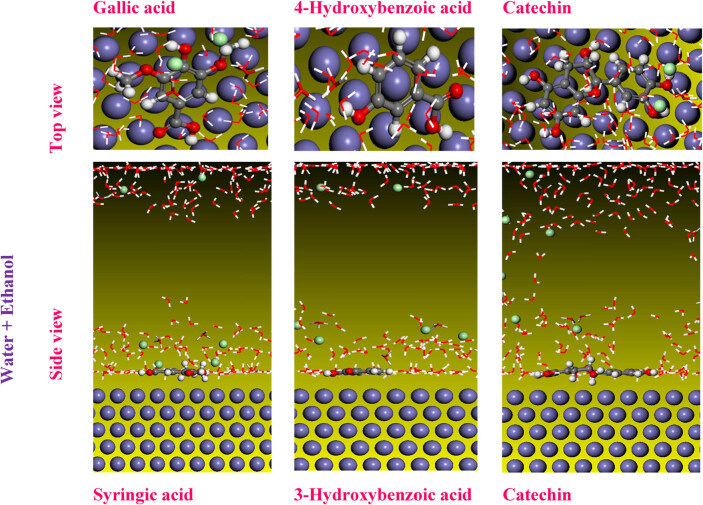
Top and side views of the stable configuration for the studied molecules in the DFT study.

A MC simulation was performed on Fe 110/200 H_2_O systems to enhance our comprehension of how molecular structures interact with the iron surface. The most stable adsorption configuration of the inhibitors under investigation is illustrated in Figure 11, presenting both a top view and a side view.

The inhibitory performance of the examined chemical structures has previously conducted, appearing that the sinapic acid structure exhibits higher reactivity compared to quercetin 3-*O*-β-d-glucoside and *p*-coumaric acid in an ethanolic extract. Moreover, gallic acid exhibits significant reactivity compared to other structures in a water extract. In contrast, syringic acid is more reactive than other structures in an IVR extract, representing a mixture of water and ethanol. The simulation interface containing the Fe 110 inhibitor and 200 water molecules shows that the structures found are adsorbed concerning the iron surface layer.

It can be observed that the absorption of *p*-coumaric acid increases with the rise in concentration, indicating a stronger interaction with the medium at higher concentrations. In contrast, in the water extract, the catechin molecule showed some inclination compared to 4-hydroxybenzoic acid and gallic acid, which can explain the high adsorption of these molecules in the steel surface. The results of the mixed extract show that catechin is also adsorbed with fewer reactive sites compared to 3-hydroxybenzoic acid and syringic acid [83].

Furthermore, based on the absolute value of *E*_ads_, the adsorption energy follows the following order: sinapic acid > quercetin 3-*O*-β-d-glucoside > *p*-coumaric acid in the ethanolic IVR extract. In the water IVR extract: gallic acid > 4-hydroxybenzoic acid > catechin. Finally, syringic acid > 3-hydroxybenzoic acid > catechin in the mixed IVR extract. This order validates the results of the DFT calculations (Table 9). These results indicated that the adsorption energy of water is very low compared to the other molecular forms studied, which allows us to conclude their migration from the surface of iron [74].

**Table 9:** MC simulation parameters

Solvant	Molecules	*E*_ads_	Inhibitor: d*E*_ad_/d*N*_*i*_	Rigid adsorption energy	Deformation energy	H_2_O: d*E*_ad_/d*N*_*i*_	H_3_O^+^: d*E*_ad_/d*N*_*i*_	Cl^−^: d*E*_ad_/d*N*_*i*_
Ethanol	Sinapic acid	−3855.70	−158.51	−3990.86	135.16	−12.86	−147.40	−143.63
	Quercetin 3-*O*-β-d-glucoside	−3781.43	−210.18	−3919.55	138.12	−11.87	−126.26	−138.87
	*p*-Coumaric acid	−3759.53	−119.11	−3890.15	130.62	−10.38	−147.85	−152.96
Water	Gallic acid	−3713.40	−118.20	−3853.04	139.64	−8.05	−138.61	−146.11
	4-Hydroxybenzoic acid	−3666.35	−104.90	−3801.91	135.56	−12.72	−143.30	−163.31
	Catechin	−3490.41	−192.30	−3620.79	130.37	−12.91	−148.52	−124.30
Water + ethanol	Syringic acid	−3748.77	−144.59	−3886.1	137.33	−13.37	−149.59	−153.08
	3-Hydroxybenzoic acid	−3709.56	−102.36	−3845.48	135.92	−12.14	−149.03	−149.90
	Catechin	−3490.41	−192.30	−3620.79	130.37	−12.91	−148.52	−124.30

## 4 Conclusion

The corrosion of materials remains a significant global issue, with substantial costs and implications for material longevity. Large corporations are actively seeking effective and sustainable solutions to mitigate or reduce material corrosion. In this scientific study, we have demonstrated that the roots of the *I. viscosa* plant, found in the Moulay Yaâcoub province of Morocco, can efficiently inhibit corrosion in various extracts, particularly in the mixed extract composed of water and ethanol, achieving a remarkable inhibition rate of 96.6%. We observed that the combination of these two solvents in the IVR extract yields excellent corrosion inhibition efficiency. Analyses conducted using EDX and SEM techniques on the various studied extracts have revealed a significant reduction in the dissolution of MS, owing to the formation of a protective and preservative surface layer. Additionally, HPLC analyses identified three major molecules in each studied extract. Notably, HPLC results for the mixed extract enabled the identification of three predominant molecules: syringic acid, 3-hydroxybenzoic acid, and catechin, which contribute to outstanding corrosion inhibition compared to other studied extracts. Therefore, considering the adsorption constants, we suggest that the adsorption phenomenon of the studied inhibitors can be better represented by the Langmuir model for different extracts. It should be noted that the Langmuir model exhibits a very high correlation coefficient of 0.99. Finally, the application of DFT and classical MC simulation methods has established a strong correlation between experimental data and theoretical predictions, thereby enhancing our understanding of the underlying mechanisms.
